# Anticancer properties of *Tulbaghia violacea* regulate the expression of p53-dependent mechanisms in cancer cell lines

**DOI:** 10.1038/s41598-020-69722-4

**Published:** 2020-07-31

**Authors:** Lesetja R. Motadi, Mpho S. Choene, Nonkululeko N. Mthembu

**Affiliations:** 1 Department of Biochemistry, Faculty of Science, University of Johannesburg (Kingsway Campus), P.O. Box 524, Auckland Park, 2006 South Africa; 2 Department of Consumer Science, University of South Africa (Florida Campus), Private Bag 1, Pretoria, 0001 South Africa

**Keywords:** Cancer epigenetics, Apoptosis

## Abstract

Cancer is an enormous burden of disease globally. Today, more people die from cancer than a combination of several diseases. And in females, breast and cervical malignancies remain the most common types. Currently, cervical and breast cancer are the most diagnosed gynecological cancer type amongst black females in the Southern Sahara while amongst males prostate cancer is on the upward trend. With many of them still dependent on medicinal plants as a form of therapy and the need to identify new therapeutic agents, we have identified a commonly used medicinal plant *Tulbaghia violacea* Harv. commonly known as Itswele lomlambo (Xhosa), wilde knoffel (Afrikaans) and Isihaqa (zulu) to evaluate its anticancer properties at a molecular biology level. In this study, we evaluated the molecular mechanism of *T. violacea* extracts in regulating cell death in various cancer cell lines. To achieve this, *T. violacea* was collected, dried before crushing into a fine ground powder. Three organic solvents namely, methanol, hexane, and butanol at 10 g per 100 mL were used as extraction solvents. Each cell line was treated with varying concentrations of the plant extract to identify the half-maximal inhibitory concentration (IC50). The IC 50 was later used to analyse if the extracts were inducing apoptosis using annexin V analysis. Furthermore, the molecular mechanisms by which apoptosis was induced was analysed by qPCR, western blots. All three extracts exhibited anticancer activity with the most cytotoxic being methanol extract. p53 expression was significantly increased in treated cells that correlated with increased caspase activity. The results point to possible activation of apoptosis following treatment with hexane extracts.

## Introduction

Cancer is a major cause of death worldwide. Surgery, chemotherapy, and other cancer treatments have been known to reduce cancer-related deaths^1^. Current drugs or other approaches to counteract chemotherapy-induced adverse effects are often incompletely effective, frequently do not address potential longer-term sequelae or may even induce other side-effects which only add to patient discomfort. Thus, much research is now being carried out to search for better chemotherapy drugs from naturally occurring compounds that can suppress or prevent the process of carcinogenesis^2,3^. It is a widely recognized fact that numerous synthetic chemotherapeutic drugs exert positive effects but also harmful side effects such as cardiotoxicity and hair loss associated with vomiting. In contrast, in plants, strongly active substances coexist with other compounds that antagonize each other. Therefore, there has been a renewed call by the World Health Organization (WHO) to screen plant material for the presence of biologically active compounds. A successful anticancer drug should kill cancer cells without causing unnecessary damage to normal cells, and that can only be achieved by restoring the apoptosis machinery in cancer cells^4^. Several, studies have previously demonstrated the cytotoxic activities of extracts of *T. violacea* (Wild garlic from Southern Africa) in cancer cells^5^. The life span of both normal cells and cancer cells is extensively affected by the rate of apoptosis. Thus, modulating apoptosis may be useful in the management and therapy or prevention of cancer. Significantly, natural products provide such templates^6,7^. Thus, it is imperative that apoptotic inducers be screened from plants, either in the form of crude extracts or as compounds isolated from them^8^. Therefore, in this study, we evaluated the apoptotic induction potential of *Tulbaghia violacea *(TV) in various cancers.

## Experimental

### Cell culture

All cell lines used in this study were purchased from Japanese Health Science Foundation. Cells were grown in their respective medium at 37 °C in a humidified atmosphere with 5%, CO_2_-95% air. The cells were trypsinized (0.1% trypsin) once reached 85% confluency and counted, plated in 96 well plates for treatment with desired extracts.

### Preparation of the herbal extract

*Tulbaghia violacea* leaves were collected from parts of the Western Cape and Kwazulu-Natal provinces, South Africa. The plant species were analysed at Parceval pharmaceuticals and the following voucher number was provided PAR-TU-VIO-002. The dried materials were ground to a fine powder by a mechanical grinder. The powdered leaves were soaked (1 g in 10 ml of solvent) at room temperature overnight shaking. The extract was filtered through whatman paper no.40 and the resultant filtrate was evaporated under negative pressure using a rotary vacuum evaporator. The following equation: Yield (g/100 g) = (W1 × 100)/W2 where W1 is the weight of the extract residue obtained after solvent was used.

The extraction yield (%) was calculated as follows^9^:

$${\text{Extract}}\;{\text{yield }}\% = \frac{{{\text{Weight}}\; {\text{of}}\; {\text{the}}\; {\text{extract}}\; {\text{after}}\; {\text{evaporating}} \;{\text{solvent}} \;{\text{and}} \;{\text{freeze}} \;{\text{drying}} }}{{{\text{Dry}}\; {\text{weight}} \;{\text{of}}\; {\text{the}} \;{\text{sample}}}} \times 100$$

### UPLC analysis

A Waters UPLC coupled in tandem to a Waters SYNAPT G1 HDMS mass spectrometer was used to generate accurate mass data. Optimisation of the chromatographic separation was done utilising a Waters BEH C8 column and temperature controlled at 60 °C. A binary solvent mixture was used consisting of water (Eluent A) containing 10 mM formic acid (natural pH of 2.3) and acetonitrile (Eluent B) containing 10 mM formic acid. The initial conditions were 98% A at a flow rate of 0.4 mL/min and were maintained for 1 min. The runtime was 30 min and the injection volumes ranged between 1 and 3 µL.

### Cell viability analysis

Cell viability was determined using an MTT assay. About 10,000 cells were plated per well in a 96-well plate. Cells were grown for 24 h at 37 °C this was followed by replacement of media containing different extracts concentration. After 24 h 20 µL of 5 mg/mL MTT was added to each well. Cells were incubated for 3.5 h at 37 °C in a 5% CO_2_ culture. The media and MTT were removed and 150 µL of DMSO was added. Cells were agitated on an orbital shaker at 75 rpm for 15 min before absorbance was read at 590 nm with a reference filter at 620 nm.

### xCELLigence assay

Before the start of the experiment, the xCELLigence instrument was placed in a 37 °C incubator than a volume of 100 µL of antibiotic-free culture medium was added onto the 16-well E-Plate and the plate was placed on the xCELLigence instrument to record the background reading. 1.5 × 10^4^ cells were seeded onto the well plates and the E-plate was placed back to the current flow of the instrument. The next day, cells were treated with 20 µM of methanolic extract of Tv and cell growth monitored for 48 h. Cell index values were recorded at 15 min' interval sweeps until the end of the experiment. The xCELLigence parameters were as follows: Step 1: 1 sweep, 1 min, 00:00:01 total time to measure background. Step 2: 100 sweeps, 15 min' interval, 24:45: 01 total time to measure cell impedance. Step 3: 25 sweeps, 15 min' interval, 30:45:01 total time.

### Apoptosis analysis

Cells of about 1 × 10^4^ cells were added onto a 6-well plate containing coverslips. The plate was incubated overnight to allow the cells to attach onto the surface. Following attachment, cells were treated with media containing IC_50_ of plant extracts for 24 h. Four percent (4% of Formaldehyde) was added onto each slide/cover slide and the plate incubated for 20 min at room temperature, to allow an efficient fixation of cells. Cells were washed twice with PBS and once with 0.1% BSA wash buffer and further stained with DAPI and Annexin V/FITC for 5 min. BX-63 Olympus microscope (Germany) was used to visualize the cells.

### Caspase 3/7 analysis

For analysis of caspase activity, 100 uL 1 × 10^4^ cells were plated overnight on a 96-well luminometer plate and allowed to attach overnight. The cells were treated with different IC_50_ concentrations of plant extracts and incubated for a period of 24 h. Caspase-Glo 3/7 assay was performed according to manufacturer’s protocol (Promega, USA). Briefly, following treatment, media was replaced with caspase glo 3/7 reagent mixed with a substrate at a ratio of 1:1 *v*/*v* of DMEM: Caspase-glo 3/7 reagent and was incubated for 2 h at 37 °C in 5% CO_2._ Luminescence was quantified using GLOMAX from Promega (USA). The assay was conducted in triplicates and caspase 3/7 activity was reported as a mean of Relative Light Units (RLU). The following formula was used to calculate caspase 3/7 activity in RLU.

### Gene analysis using qPCR

RNA was extracted using Nucleospin® RNA II total RNA isolation kit according to the manufacturer's protocol and quantified using a nanodrop (NanoDrop Technologies, USA). Following RNA extraction, cDNA was synthesized using ImProm-II™ Reverse Transcription system from Promega®. qPCR was then performed in a 20 μL reaction mixture containing 2 μg/μL cDNA, SYBR Green (SIGMA ) and primers under the following conditions: 36 cycles of 94 °C for 30 s, 60 °C for 30 s, and 72 °C for 30 s.

### Western blot analysis

Following 24 h of treatment with IC_50_ concentrations, cells were lysed using RIPA buffer (50 mM Tris–HCl pH 7.4, 150 mM NaCl, 1% NP-40, 0.1% SDS, 2 mM EDTA). Protein content was measured by the BCA assay and equal amounts were electrophoresed in SDS polyacrylamide gel and then transferred onto nitrocellulose membranes. Membranes were subsequently immunoblotted with Anti-mouse monoclonal antibodies used at 1:500–1000 dilutions as primary antibodies, while a goat anti-mouse horseradish peroxidise-conjugated horse IgG (Santa Cruz, USA) was used at a 1:2000 dilution as a secondary antibody. The membranes were developed using Chemiluminescence detection kit (Santa Cruz Biotechnology, CA). The membranes were imaged using a Biorad ChemiDoc MP.

#### Data analysis

Experiments were performed in duplicates. Statistical analysis of the graphical data was expressed as the mean standard deviation. The *p* value was analysed in comparison to the untreated using Student *t*-Test wherein *p* < 0.05 was considered as significant.

## Ethical approval

This article does not contain any studies with human participants or animals performed by any of the authors.

## Informed consent

For this type of study, formal consent is not required.

## Results

This section presents the observations that we have noted following treatment of cancer cells with varying concentrations of the extract and then followed by the verification of cell death.

### UPLC-QTOF-MS profiling

The organic extracts are composed of intermediate-polar secondary metabolites.

**Figure 1:**
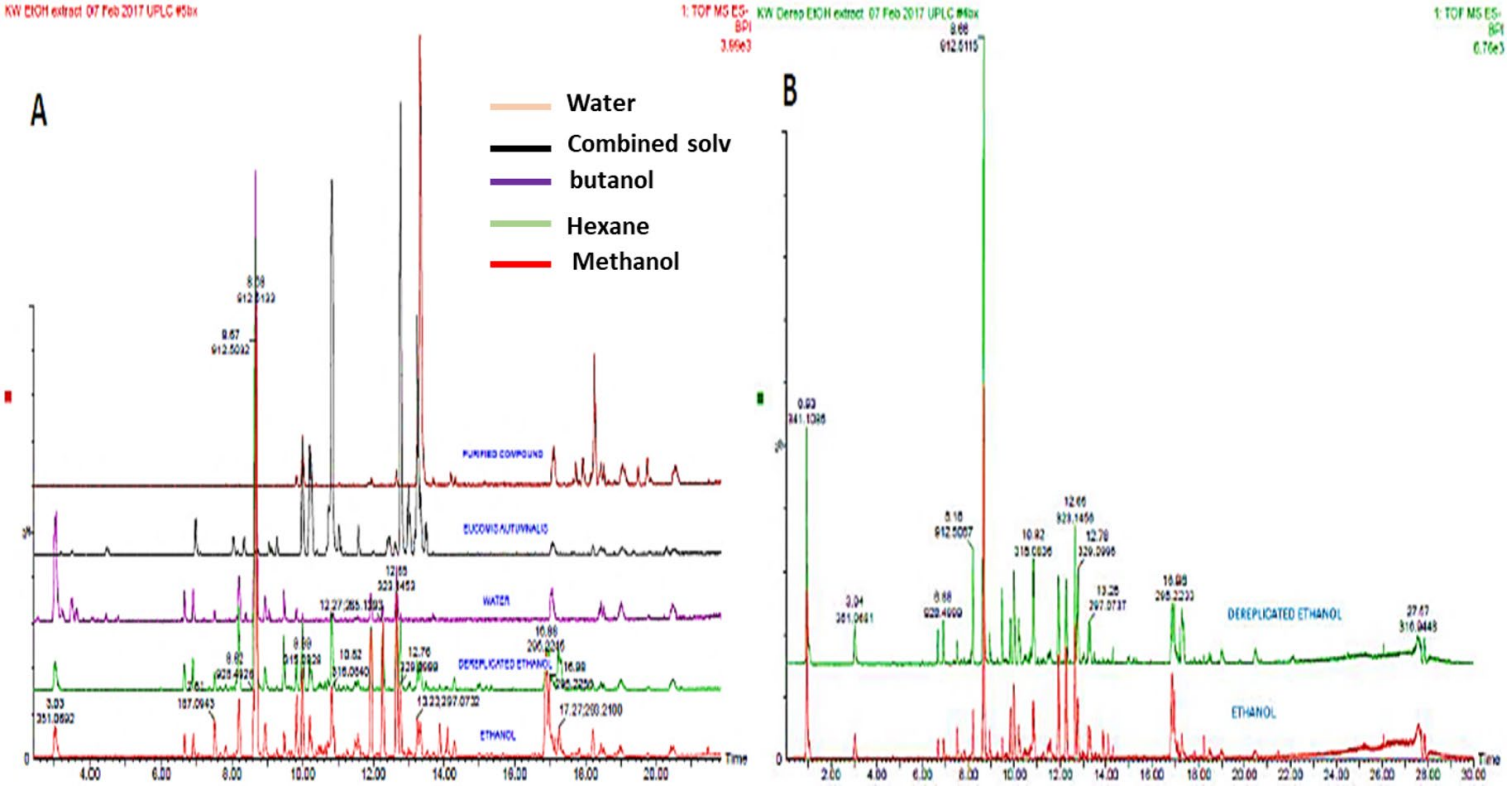
Represented UPLC-QTOF/MS chromatograms of the South African herbal concoction. (**A**) overlaid chromatograms of all samples including the extract of *Tulbaghia violacea* and (**B**) overlaid chromatograms of dereplicated hexane versus methanol extract; (All chromatograms were monitored in negative electro-spray ionization mode (ESI^-^). The y-axis shows the percentage intensity of the peaks while the x-axis shows the retention time of the corresponding peaks.

To evaluate the effect that solvents have on the type of metabolites extracted during herbal extract preparation, we prepared both hexane and methanol extracts. The Fig. 1B depicts visual examination of the Base Peak Intensities (BPI) of the chromatograms in the negative electro-spray ionization (ESI^-^) mode of the different herbal extracts. The chromatograms indicate clearly that the methanol extract has different phytochemicals/metabolites when compared to the hexane extract. Therefore, as expected, we can see that the different solvents used for extraction, induced differential secondary metabolite changes as demonstrated by increases or decreases in peak intensities and the appearance of new molecular ion peaks and suppression of other molecular ion peaks, which are present in the BPI’s of other chromatograms. All three extracts indicate that they possess some similar metabolites as seen from extract peaks aligning (Fig. 1A).

### Cell viability analysis using MTT

**Figure 2:**
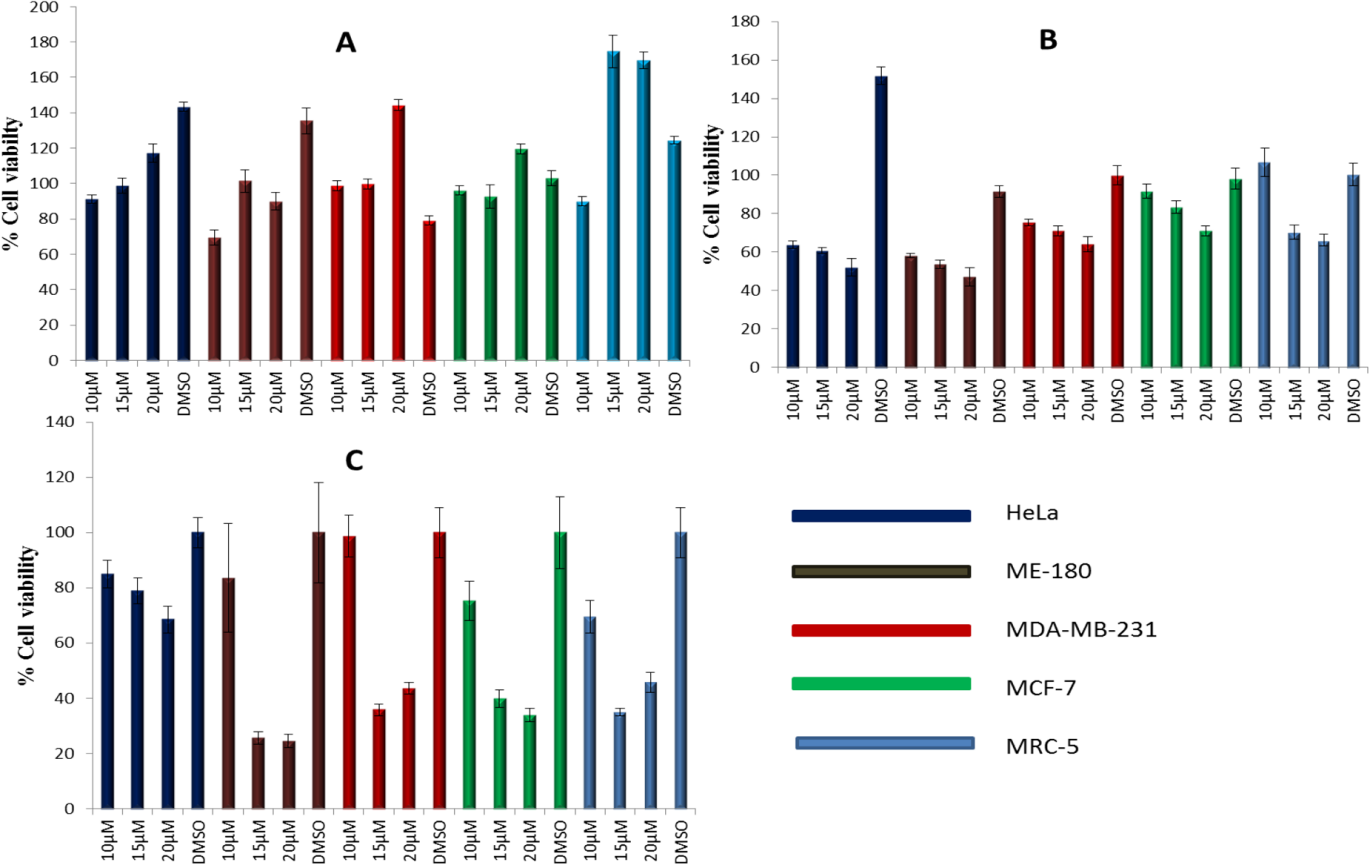
Cell viability analysis using MTT. Cell were treated with varying concentrations (10, 15, 20 µM) of the (**A**) butanol, (**B**) hexane and (**C**) methanol extracts of Tulbaghia voilacea (TV) respectively. HeLa, ME-180, MDA-MBA-231, MCF-7 and MRC-5 cell lines were treated with the plant extracts for 24 h. Cell viability was determined in triplicate from three independent experiments by MTT assay. Data represented as mean ± SD, **p ˂ 0.10, *p ˂ 0.05.

All cell lines were treated with varying concentrations of butanol (A), hexane (B) and methanol(C) *Tulbaghia violacea *(TV) extract for 24 h. The concentrations were as follows DMSO 0.05%, 10 µM, 15 µM and 20 µM as reported in the results. However a lower concentration of 5 µM and a higher 30 µM were used but due to their either ineffectiveness or high toxicity they were dropped and only concentrations that were also applied on xCELLigence RT cell viability assay were considered for a perfect IC50 prediction. IC_50_ was characterised as concentrations where 50% of cell death was observed following treatment with different concentrations. The results indicated that inhibition of cell proliferation by *T. violacea* extracts was in a dose-dependent and cell line dependent manner. The methanolic extract exhibited the highest cytotoxicity of the three extracts, with ME-180 cells exhibiting the most sensitivity at 25% toxicity at both 15 µM and 20 µM. The butanol extract did not significantly affect the viability of all of the cell lines tested up to concentrations of 20 µM. HeLa cell lines appeared to exhibit the highest resistance of the five cells lines to the butanolic and methanolic extracts where the highest concentration tested (20 µM) failed to induce half-maximal inhibitory concentration (IC_50_) (Fig. 2). Both HeLa and ME180 which are cervical cancer cells showed potency in 15 µM and 20 µM of about 55% and 49% respectively. MTT assays is an endpoint assay and can only detect cell viability/ cytotoxicity at a particular time point. The preferred IC50 concentration was found to be 15 µM. Hence, further performing experiments would confer a better understanding of the kinetics of cell and plant extract interactions over an extended period.

### xCELLigence cell proliferation analysis

**Figure 3:**
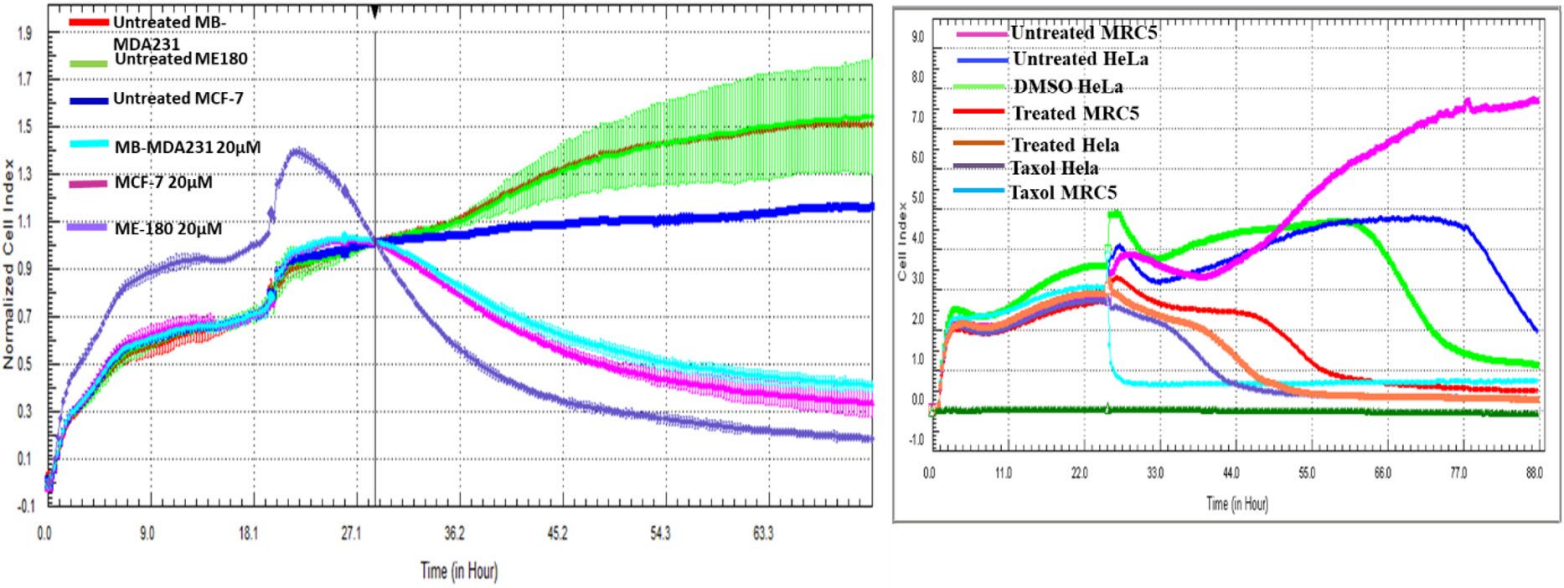
Cell Proliferation of MB-MDA231, ME180, MRC5, HeLa and MCF-7 following treatment with methanolic extract of Tv. Growth of untreated cells MB-MDA231 (red), Untreated ME-180 cells (green), Untreated MCF-7 cells (blue), MCF-7 cells treated with 15 µM of methanolic extract of Tv, MB-MDA Cells Treated with 20 µM (light/sky blue) and ME-180 cells treated with 20 µM (purple) were monitored in real time over a period of ~ 72 h. The experiments were done in duplicates and two independent biological repeats (n = 2).

From the results obtained in MTT, the treatment of the following cells have shown interesting and contradicting results MB-MDA231, MCF-7, HeLa, MRC5 and ME-180. Because of this contradictory, we have opted to observe cell proliferation over a period of 72 h using xCELLigence machine. The other cell lines were also monitored but presented results that did not show any contradictory to MTT. As shown in Fig. 2, there was a reduction in cell growth in response to 15 µM methanolic extract in MB-MDA231, MCF-7 and ME-180 all cells showed a significant reduction in cell proliferation over time. Whereas those that were untreated continued to grow even after 48 h of plating (Fig. 3).

### Caspase 3/7 activity analysis

**Figure 4:**
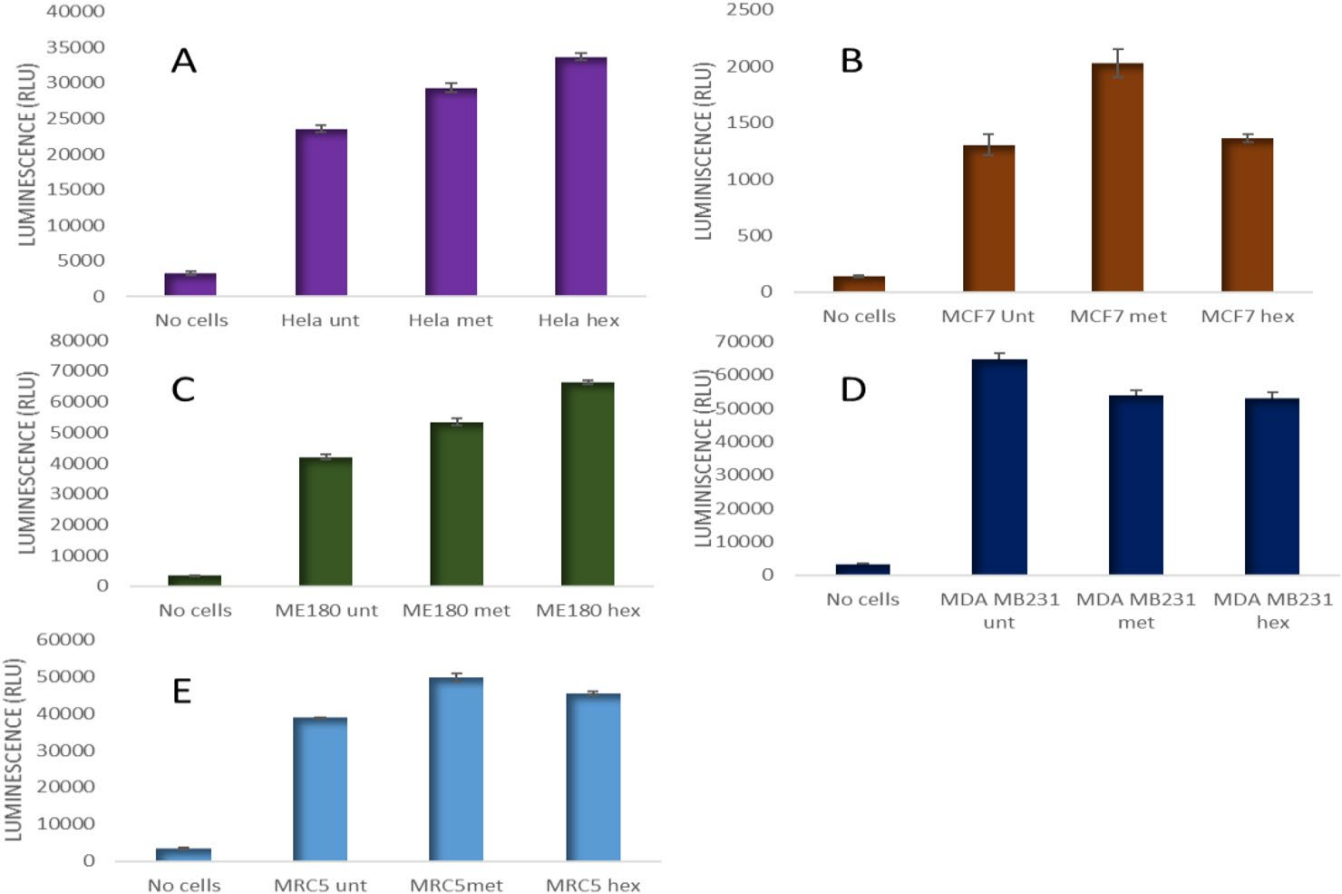
Caspase 3/7 activity (RLU) in cancer cells treated with methanol and hexane extracts of *T. violacea*. (**A**) Hela, (**B**) MCF7, (**C**) ME-180, (**D**) MDA-MB-231 and € MRC5 cell lines were treated for 24-h with extracts of TV. Enzymatic activities of caspase 3/7 activities in treated and untreated cells were then determined by luminescence assay. This was analyzed using one-way ANOVA with Dunnett's comparison post hoc test, *p* < 0.05.

Since the hexane and methanol extracts exhibited significant cytotoxicity on the cell lines, a further test we then carried out using these two extracts. To confirm if the induced cytotoxicity was due to apoptosis caspases 3/7 activity was analysed on cancer cells following treatment with TV extracts. Cells were treated with IC50 of TV and analyzed to detect for the activity of caspase 3/7 (Fig. 4). As expected, Hela and ME-180 cell lines treated with both methanol and hexane TV extract showed an increased caspase 3/7 activity, with the highest activity recorded for the hexane extract. With MCF-7 cell, an increase in caspase 3/7 activity was observed when treated with methanol extract, however, reduced caspase 3/7 activity could be observed when treated with the hexane extract. A slight reduction in caspase 3/7 activity was observed in MDA-MB-231 treated with both the methanol and hexane extracts of TV. In MRC5 cell lines, there was no significant increase in caspase activity treated with either methanol or hexane of TV.

### Microscopic analysis of apoptotic characteristics following treatment with TV

**Figure 5:**
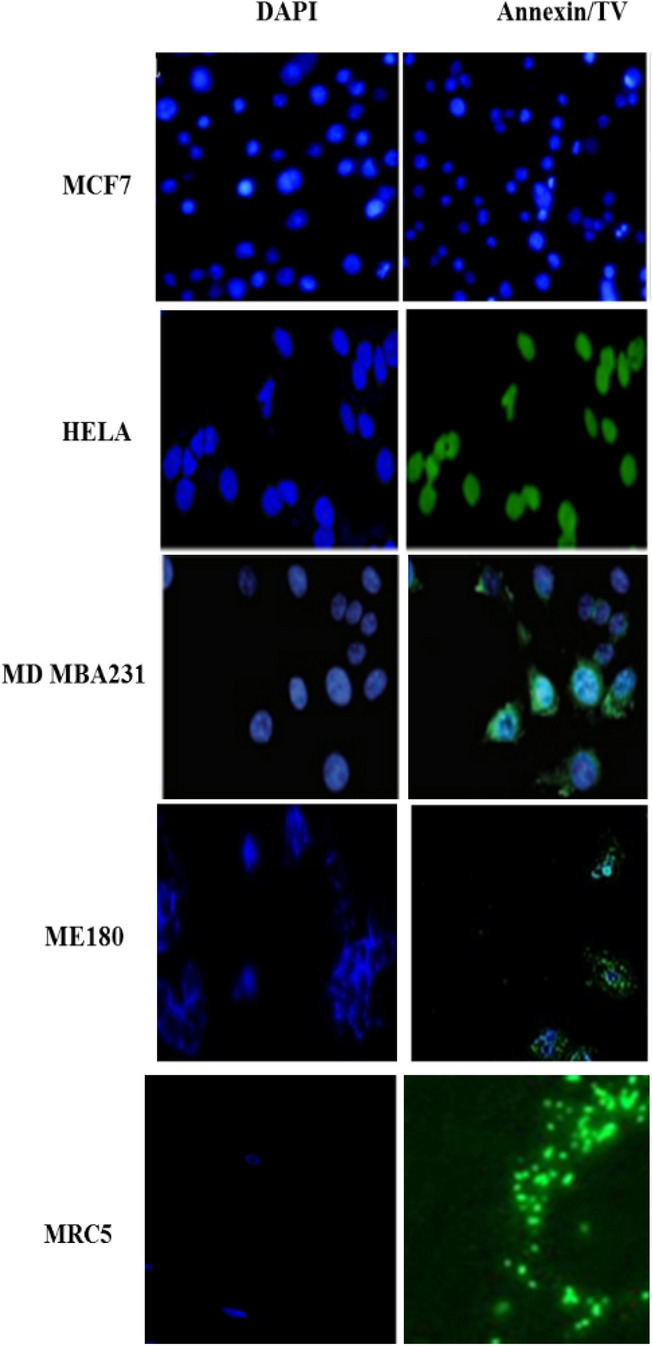
TV methanolic extract induces apoptosis in cancer cells. The results show morphological changes following treatment of cancer cell with 15 µM of methanolic extract. As earlier shown in caspase 3/7 activity, MD MBA231, HeLa and ME180 treated with IC50 showed apoptotic bodies with some cells showing shrinkage and condensation which are typical apoptotic morphological characteristics. In each panel, the bottom quadrant contains apoptotic cells that are positive for Annexin V. except MCF-7 that showed no positive staining.

One of the main characteristics of apoptosis remains the shrinking of cells and they staining positive for annexin V. As earlier shown in caspase 3/7 activity, MD MBA231, HeLa and ME180 treated with IC50 showed apoptotic bodies with some cells showing shrinkage and condensation which are typical apoptotic morphological characteristics. ME180 further shows evidence of shrinking cells with some apoptotic bodies. Little apoptotic bodies can be seen in MCF-7 and MRC 5 cell stained with Annexin V (Fig. 5).

### Real-time PCR analysis of cell cycle regulatory genes

**Figure 6:**
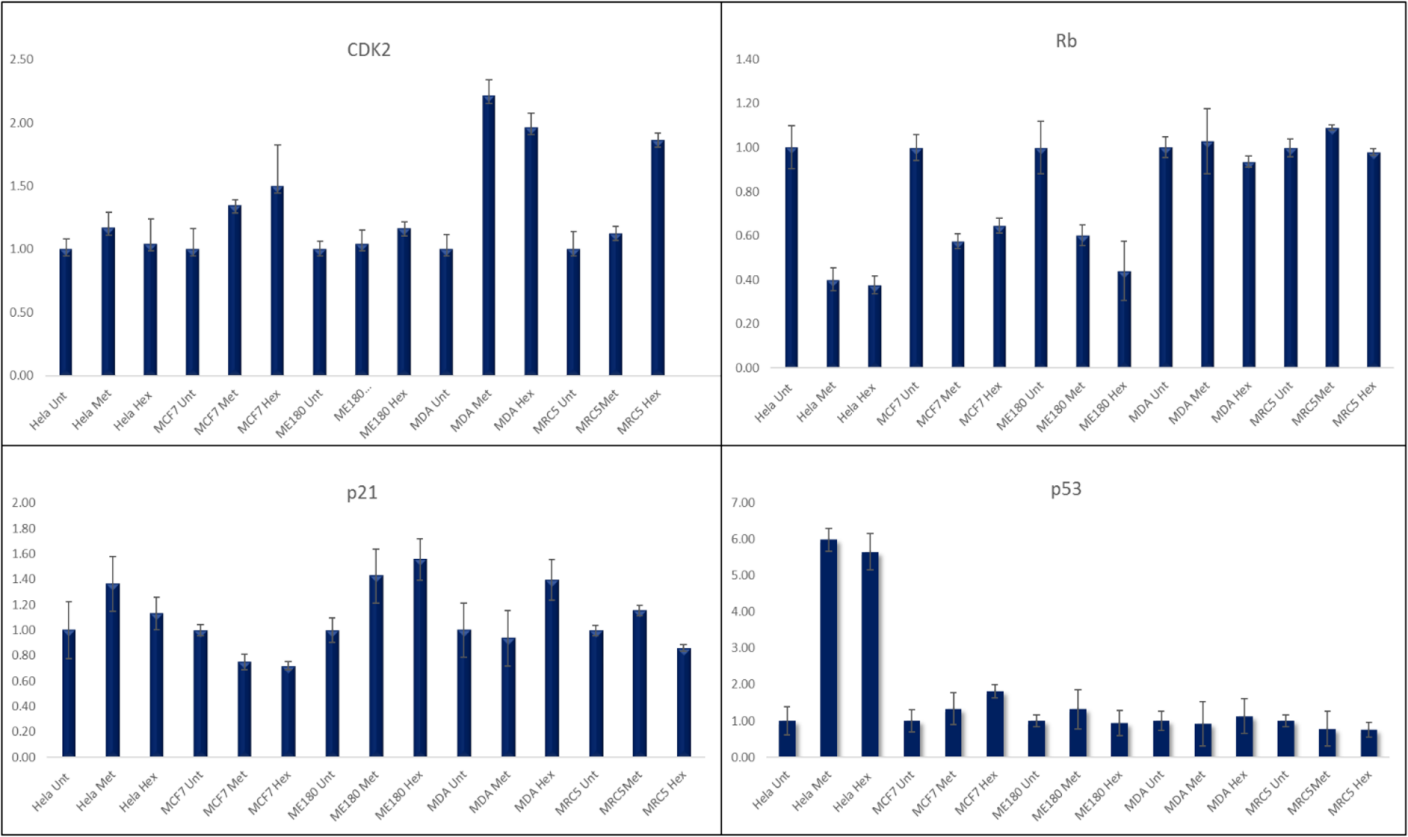
Real time PCR analysis of cell cycle associated genes following treatment with TV plant extracts.

Cell cycle plays a crucial role in monitoring cell proliferation and homeostasis of cell numbers in each organ. Real-time PCR was conducted to assess the expression levels of various cell cycle regulatory genes following treatment with hexane and methanol TV extracts. A significant increase of about tenfold in p53 expression could be observed in HeLa cells treated with both extracts. A significant increase could also be observed in ME-180 cells treated with the methanolic extract. An increase in p21 could be observed in ME-180 cells treated with both extracts, however, in MDA-MB-231 cells a p21 increase only occurred in cells treated with the hexane extract. There were no significant changes in expression levels of CDk2 except for an increase in MDA-MB-231 cells treated with both the hexane and methanol extracts. A reduction in Rb expression could be observed in Hela, MCF-7 and ME-180 cells following treatment with both extracts (Fig. 6).

### The real-time PCR analysis of apoptosis-related genes

**Figure 7:**
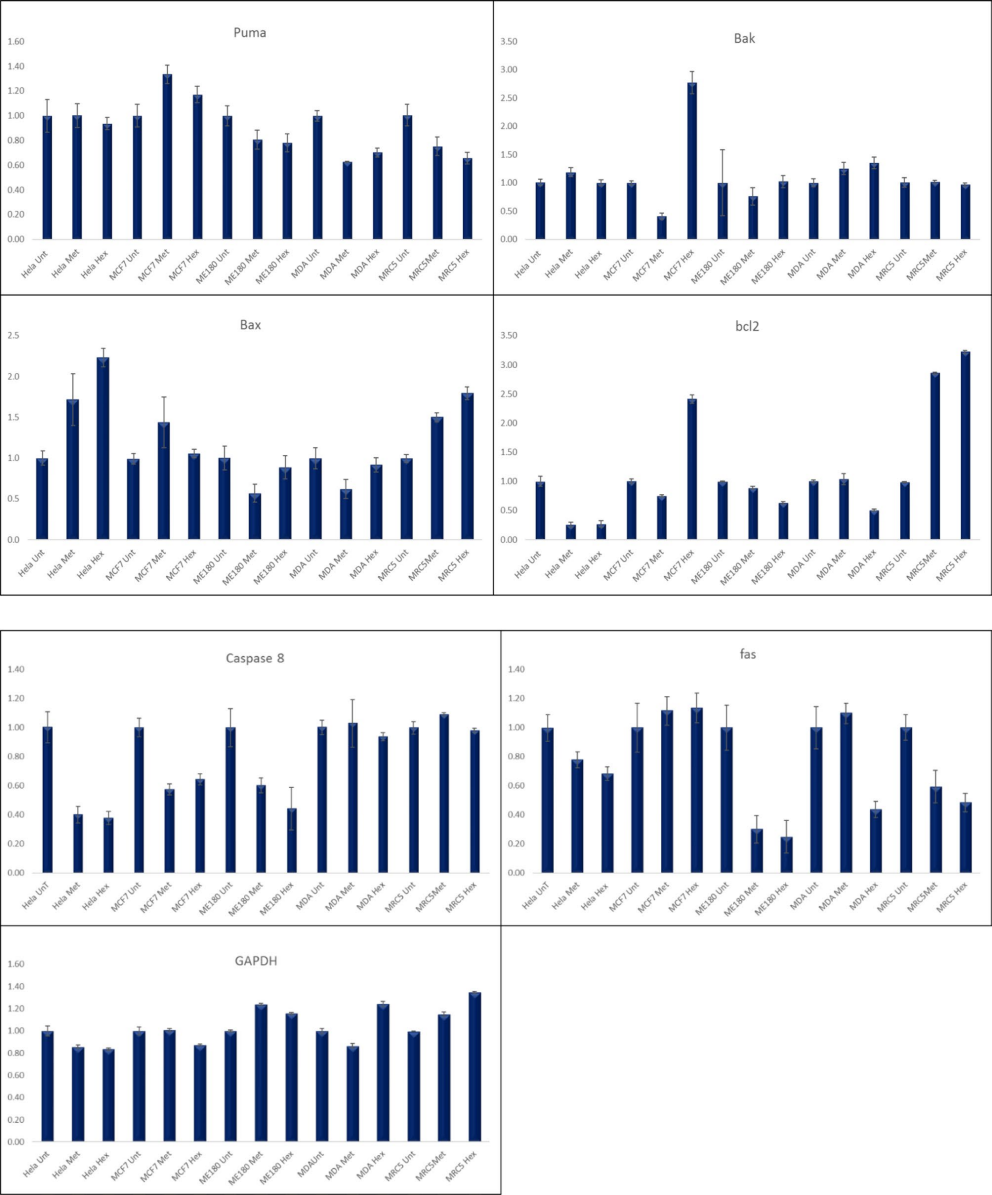
Real time PCR analysis of apoptosis related genes following treatment with TV plant extract.

The results show the different cells treated with plant extracts. From apoptosis-related genes, we observed no significant change in many of them except Bak which showed a significant increase in MCF-7 treated cells with the hexane extract from 0.8 to about 2.3 fold while Bax showed a significant increase in HeLa from 2.2 to about sevenfold increase in hexane. A change from 6.3 to about onefold increase could be observed in the MCF7 cell line treated with the methanol extract of TV (Fig. 7).

### Protein expression analysis

**Figure 8:**
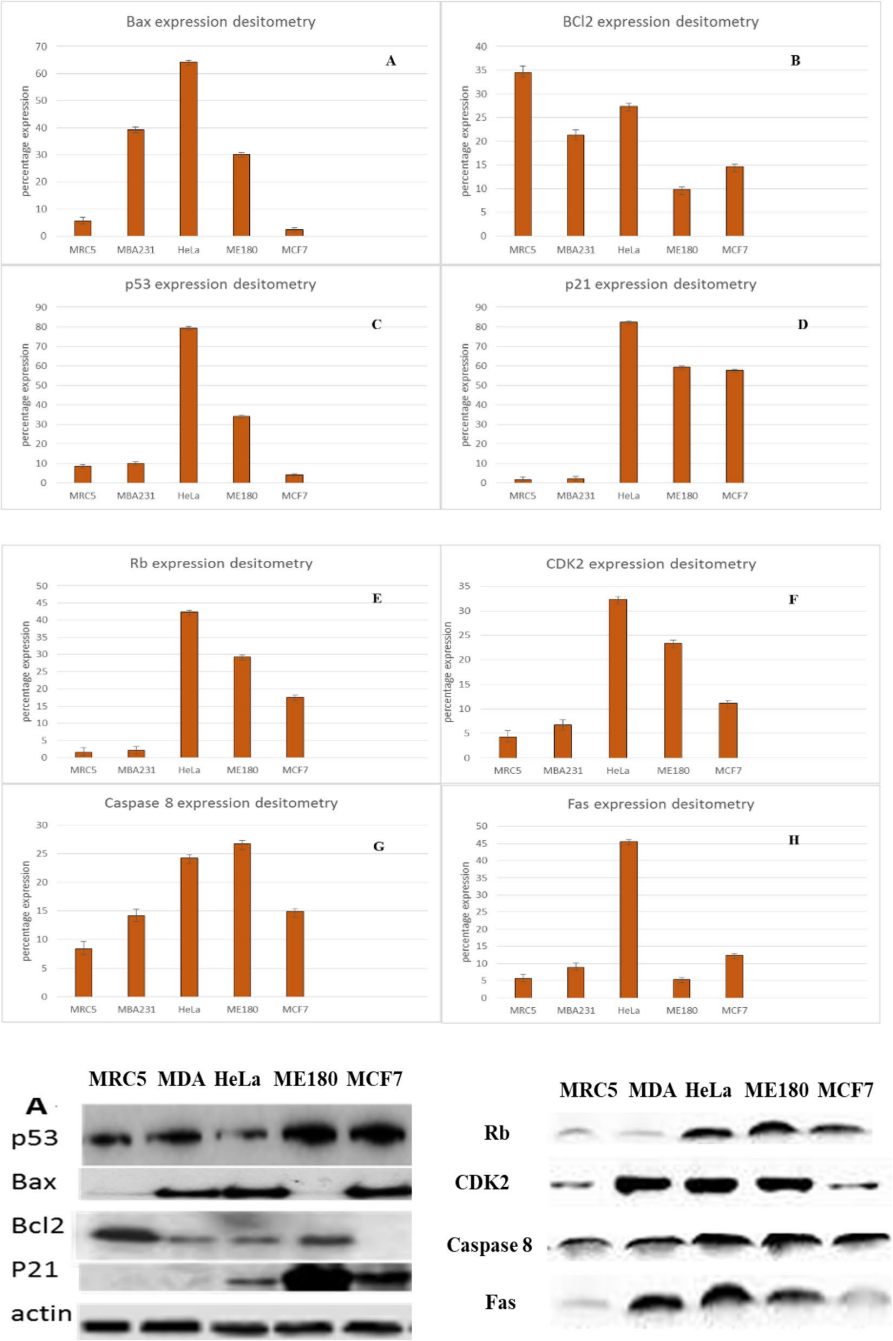
Western blot analysis of p53, bax, bcl-2 and p21 expression in several human cancer cell lines and MRC5 fibroblast following treatment with the methanolic extracts of *T. violacea*. (**A**–**H**) Protein quantification of the western blot results shown in (**A**). Protein levels are shown relative to the untreated control cells.

There earlier results predicted potential cell death by apoptosis, however it is documented that p53 plays a major role in apoptosis induction. Since methanolic extract showed apoptotic activity, molecular mechanism associated with Tv treatment was exploited. The results showed that the methanol extract of TV had the greatest effect on p53 in all cancers. In MRC5 fibroblasts, which represent normal cells, p53 was upregulated 30% following treatment. MCF7 p53 expression following treatment with TV was the lowest at approximately 12.5%, whereas HELA and ME180 p53 expression levels were the most highly upregulated at 40 and 33%, respectively (Fig. 8C).

Bcl-2 and Bax are important signalling factors of the mitochondria-mediated pathway of apoptosis that regulate cytochrome c release, activation of caspases and DNA fragmentation. Bcl-2, a member of the Bcl-2 family, is an antiapoptosis protein that promotes cancer cell growth, whereas Bax, another constituent of the Bcl-2 family, acts as a pro-apoptotic factor by inducing apoptosis. Several studies have shown that the expression of pro-apoptotic proteins such as Bax or Bak is necessary to induce cellular apoptosis and correct uncontrollable cancer growth. The ratio of BCL-2/bax determines the sensitivity of cancer cells to death signals. Alterations in Bcl-2 family protein expression have been associated with many cancer types, whereas Bcl-2 overexpression in cancer cells is regarded as a common attribute. Several studies have shown the effects of medicinal plants in downregulating Bcl-2 protein expression.

To determine the effect of methanolic TV extract, total cellular protein, including BCL-2 and Bax, was extracted and subjected to western blot analysis. As shown in Fig. 5, methanolic extracts of TV reduced Bcl2 expression in favour of Bax in MDA-MB-231 cells to a ratio of Bcl2/bax 0.0.52 with a 38% increase in Bax expression.

p21 is a universal inhibitor of cyclin-dependent kinases. By forming a complex with cyclin, cyclin-dependent kinase (cdk), and proliferating cell nuclear antigen (PCNA), it inhibits the transition between G1 and S phase^5^. Growth inhibition of both cancer cell lines and normal diploid fibroblasts has been observed. DNA damage and subsequent induction through p53 is only one possible mechanism of p21 activation. Since we showed interest in p53 activation and analysis in this study, we felt it necessary to also expand our studies to p21, as it is part of the downstream activations of p53. In this study, we observed an 81% increase in the expression of p21 in HeLa cells treated with methanolic extracts of TV. In ME180 and MCF7 cells, there were also increases in p21 expression, which were 59% and 58%, respectively (Fig. 8D). CDK2 expression was most highly upregulated in HeLa cells compared with any other cells, and CDK2 expression was similar to that of Rb (Fig. 8E, F). Apoptosis activation can either be intrinsic or extrinsic, so we evaluated the expression of Fas and caspase 8, which are mainly involved in the activation of the extrinsic pathway; we observed a significant increase in Fas in HeLa cells but a smaller increase in all other cell lines, while caspase 8 was steadily increased in all cell lines (Fig. 8G, H). As in many other studies, actin was used as our housekeeping gene, and from the results above, we observed no significant change in the expression of actin across all the treatments. This suggests that the extracts had little impact on the housekeeping gene.

## Discussion

In the present study, we evaluated the anticancer effects of *Tulbaghia violacea* in several cancer cell lines, including breast cancer (MCF7 and MB MDA231), cervical cancer (HeLa and ME-180), and in noncancerous MRC5 cells. During the study, we employed techniques that focus on the mechanism of anticancer activity of the two plants.

It is becoming increasingly clear that new therapeutic strategies against those cancers will mediate the induction of apoptosis or cell cycle. It is also evident that medical sciences have shifted searches from conventional toxic drugs to those that are synthesised by natural sources such as plants. In the present study, extracts from 3 different plants were used; however, only one was considered in this manuscript: *Tulbaghia violacea.* In all three solvents used, i.e., methanol, butanol, and hexane, there were mixed results, as shown in Fig. 2. The IC50 in all the extracts seemed to support 15 µM as the optimal concentration for treating cells. However, in some cases, at a higher concentration than that, the extract seemed to support cell proliferation more than suppress it. The results were not surprising since many plants contain both carcinogens and anticarcinogens that act to antagonize one another, and if not balanced, they might tilt the cell balance from one outcome to the other^10^. As already stated, new therapies will likely be ones that induce or restore cellular homeostatic machinery. Apoptosis induction was at the centre of our study, and from the results, we observed changes in the morphology of the cells following treatment with both hexane and methanol plant extracts. The cells exhibited by shrinkage, spikes and other properties resembling DNA fragmentation; these characteristics were similar to those reported in the past as evidence that cells are undergoing apoptosis^11–15^.

Caspases are a family of protease enzymes that play essential roles in programmed cell death and inflammation. In this study, to confirm that plant extracts induced apoptosis, caspase-3/7 activity was analysed since the two are the executors of the cells, and the increased activity signal increases apoptosis. In our study, we observed increased caspase-3/7 activity in cervical cancer cells, especially in cells treated with the hexane extract, and as expected, there was little activity in MCF7 cells, as they do not express caspase-3. MDA231 cells treated with hexane extract were sensitive to caspase-3/7 activity, whereas those treated with methanol extract did not show any significant change other than a slight drop in activity. Increased caspase activity was widely reported in the previously mentioned studies, which supports the idea that the two plant extracts induce apoptosis in cancer cells. However, it also suggests that this effect is dependent on the kind of solvent used to extract the compounds. In many studies, caspase activity has been shown to be more commonly activated in methanolic extracts^16–19^.

Many in vitro and in vivo studies have shown many medicinal plants as promising for the treatment of cancer cells. Many genes affected by plant extracts remain unknown. In this study, we mainly focused on gene expression and protein expression following treatment of cells with the extracts for 48 h. Unlike other cellular effects, the effect of the extracts on protein and gene expression can only be seen or observed after 48 h. Moreover, the results showed that the plant extracts had minimal effects on genes associated with the cell cycle, as shown in Fig. 4, in which most genes were not affected. However, p21, which was directly linked to p53, was shown to be increased by treatment with hexane extract, especially in cervical cancers. Similarly, p53 was significantly increased in HeLa and ME-180 cells at both the protein and gene levels. p53 upregulation, as further supported by the increase in Bak and Bax in both cervical cancer cell lines with ATM and Puma, occurred in cancer cells treated with both methanol extract and hexane extract^20–25^. These results support the earlier observed MTT and cell morphology results and further indicate that apoptosis is caused by plant extract-induced activation of p53.

## Conclusion

*Tulbaghia violacea* extracts exerted anticancer effects in several cancer cell lines chosen in this study with morphological changes that support the induction of apoptosis. Hexane extracts of *T. violacea* have been shown to be actively involved in the induction of apoptosis; TV increases apoptosis-related gene expression in all the tested cancers, especially cervical cancers, compared with that of methanolic extract, with the butanolic extract showing little or no effect on the cancer cell lines chosen. This further shows that p53 is mainly increased in hexane and methanol extract-treated cells, suggesting that the p53-dependent pathway may be responsible for cell death.
